# Novel Approach to Improve Immunotherapy Efficacy while Mitigating Side Effects of Alzheimer's Disease. An Experimental Approach.

**DOI:** 10.1002/alz70859_107233

**Published:** 2025-12-25

**Authors:** Giulio Maria Pasinetti, Eun‐Jeong Yang, Nicole Less

**Affiliations:** ^1^ Icahn School of Medicine at Mount Sinai, New York, NY USA; ^2^ James J. Peters Veterans Affairs Medical Center, Bronx, NY USA; ^3^ James J. Peters Department of Veterans Affairs Medical Center, New York, NY USA; ^4^ Icahn School of Medicine at Mount Sinnai, New York, NY USA

## Abstract

**Background:**

Lecanemab, a human IgG1 monoclonal antibody targeting oligomer, protofibrils and fibril forms of beta‐amyloid, has been reported to reduce amyloid pathology and improve impaired cognition after administration of a high dose (10 mg/kg) of the drug in Alzheimerʼs disease (AD) clinical trials. The purpose of this study was to investigate the therapeutic efficacy and the associated molecular mechanisms of a lower dose of lecanemab (1 mg/kg) with enhanced delivery via focused ultrasound (FUS) in a mouse model of AD.

**Method:**

We used 6‐month‐old WT and 5xFAD mouse model. The FUS with microbubbles opened the blood– brain barrier (BBB) of the hippocampus for the delivery of lecanemab. The combined therapy of FUS and 1mg/kg of lecanemab was performed three times in total and each treatment was performed biweekly. Y‐maze test and immunohistochemical experimental methods were employed in this study.

**Result:**

The FUS‐mediated BBB opening markedly increased the delivery of lecanemab into the brain by approximately 10 times in the brains. The combined treatment induced significantly less cognitive decline and decreased the level of amyloid plaques in the hippocampi of the 5×FAD mice compared with lecanemab alone. Combined treatment with FUS and lecanemab activated phagocytic microglia without hemorrhage in the hippocampi of 5×FAD mice. To understand potential underlying mechanisms, we performed RNA sequencing and identified that enriched canonical pathways including phagosome formation, neuroinflammation signaling and synaptic plasticity were altered in 5xFAD mice.

**Conclusion:**

Our study elucidated that FUS‐mediated delivery of a low dose of lecanemab (1 mg/kg) decreases amyloid deposits and attenuates cognitive function deficits. We present an AD treatment strategy through the synergistic effect of the combined therapy of FUS and lecanemab.

